# Another Type of Beetle Larva of Elateridae from Kachin Amber: A Hairy Click Beetle Larva

**DOI:** 10.3390/insects17030271

**Published:** 2026-03-03

**Authors:** Joachim T. Haug, Ana Zippel, Simon J. Linhart, Patrick Müller, Yanzhe Fu, Gideon T. Haug, Carolin Haug

**Affiliations:** 1 Faculty of Biology, Ludwig-Maximilians-Universität München, Großhaderner Str. 2, 82152 Planegg-Martinsried, Germany; zippel@biologie.uni-muenchen.de (A.Z.); simon.linhart@palaeo-evo-devo.info (S.J.L.); carolin.haug@palaeo-evo-devo.info (C.H.); 2 GeoBio-Center, LMU (Ludwig-Maximilians-Universität München), Richard-Wagner-Str. 10, 80333 München, Germany; 3 Independent Researcher, Kreuzbergstr. 90, 66482 Zweibrücken, Germany; pat14789@web.de; 4 State Key Laboratory of Palaeobiology and Stratigraphy, Nanjing Institute of Geology and Palaeontology, Chinese Academy of Sciences, Nanjing 210008, China; yzfu@nigpas.ac.cn; 5 Fakultät für Biowissenschaften, Universität Heidelberg, Im Neuenheimer Feld 234, 69120 Heidelberg, Germany; gideon.haug@palaeo-evo-devo.info

**Keywords:** Cretaceous, Burmese amber, Coleoptera, Elateridae, termitophily

## Abstract

In the modern fauna, click beetle larvae are important ecosystem components, fulfilling different ecological functions. The fossil record of click beetle larvae is still scarce. Even in the very diverse fauna of the Kachin amber forest (Myanmar, Cretaceous, ca. 100 million years old), only three morphotypes of click beetle larvae have been reported so far. Here, we add a fourth morphotype, characterised by very long setae. The mouthparts indicate a predatory lifestyle. The long and quite stiff-appearing setae might have protected the larvae, for example, when hunting in termite nests, which is a strategy that some extant click beetle larvae apply. This would also imply a closer association with wood and thus a greater likelihood of preservation in amber. Here, we present twelve larvae of this new morphotype, representing two or three possible species, including an ontogenetic series for one of these.

## Simple Summary

Beetle larvae have very different functions in ecosystems today. This is also true for the larvae of click beetles. Very few fossils of click beetle larvae have been found to date. In 100-million-year-old amber from Kachin, Myanmar, a very diverse fauna is preserved, but even there, only three different morphotypes of click beetle larvae are known. In this study, we present a fourth morphotype of fossil click beetle larvae, which has very long setae on its body. The morphology of its mouthparts points to the larvae being predators. Because the setae on the body are long and apparently rather stiff, they might have protected the larvae while hunting, for example, in termite nests, which is what some click beetle larvae do today. As termites live near or in wood, this makes it more likely that the click beetle larvae will become trapped in resin, which will later become amber. Here, we present twelve of these fossilised larvae of the new morphotype, which represent two or three possible species and seem to include a developmental series for one of these.

## 1. Introduction

There are currently more than 442,000 formally described extant species of beetles (Coleoptera) [1], which shows that the group is extremely species rich. Beetles also constitute a variety of ecological functions, especially the larvae (recently reviewed in [2]). We can assume that in past faunas, they also played an important role.

Based on the available literature, beetle larvae are not as abundant in the fossil record as we would expect them to be [3]. This is likely an artefact of the tradition of focusing on adults and ignoring larval specimens [4]. Part of this tradition likely stems from the fact that larvae are often more difficult to treat from a taxonomic point of view. However, some larvae are indeed quite distinct in appearance and can, to a certain level, even be identified by non-experts. Among these larvae is the wireworm ([5] p. 410), the larval type of many click beetles. The body of these larvae is pronouncedly cylindrical and elongate; therefore, wireworms can easily be identified based on their habitus. The body shape of the latter even received its own term, namely, elateriform [6], referring to the name of the group of click beetles, which is Elateridae. However, not all larvae of click beetles develop via these typical wireworm larvae; the elongate cylindrical larvae are found, for example, in Elaterinae. Click beetle larvae have a significant impact on modern-day ecosystems and are also of economic relevance [7,8,9,10,11,12].

Distinct larvae like wireworms are also expected to be easily detected as fossils. However, at present, fossil larvae of Elateridae are rare, even in amber, which usually has a high preservation potential. Kachin amber from Myanmar has provided an astonishing amount of fossil specimens in recent years, including larvae of holometabolans, and among them, beetle larvae [13,14,15,16,17,18,19,20,21,22,23,24,25,26,27,28,29,30,31,32,33,34,35,36,37]. It is therefore not surprising that among the few cases of preserved fossil click beetle larvae, several types have been reported from Kachin amber, representing three different morphotypes [3,38,39,40]. However, the abundance and number of different types is still low.

**Figure 1:**
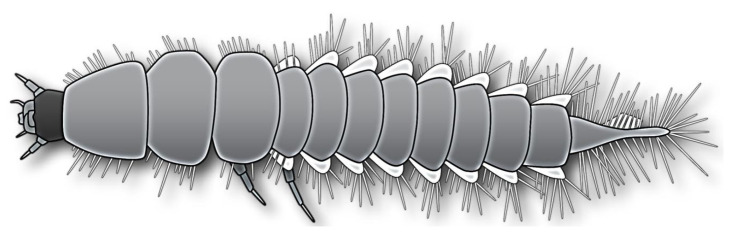
Schematic representation of the hairy click beetle larva from Kachin amber from Ref. [15].

Some books have provided a general overview of occurrences in Kachin amber, including beetle larvae [15,18]. One of these (Ref. [15] p. 117 bottom left; see Figure 1 for schematic interpretation) is an elongated larva that appears quite hairy. Here, we report additional specimens with a similar appearance and identify this type as a click beetle larva.

## 2. Materials and Methods

### 2.1. Material

In total, twelve specimens of beetle larvae preserved in eleven amber pieces were directly studied. All originate from Kachin amber, Myanmar, which has been interpreted as being of Cretaceous age [41,42]. Six specimens are part of the Palaeo-Evo-Devo Research Group Collection of Arthropods, Ludwig-Maximilians-Universität München (LMU Munich). These specimens were purchased on the trading platform ebay.com (accessed on 25 January 2026) from the trader burmite-miner. Repository numbers for these are PED 1360, 2456, 2597, 3641, 3775, and 4078.

Four pieces are part of the collection of one of the authors (PM) and stored under repository numbers BUB 3071, 3087 (containing two specimens), 3692, and 3707. One amber piece is part of the collection of the Nanjing Institute of Geology and Palaeontology of the Chinese Academy of Sciences and stored under the number NIGP209583.

### 2.2. Documentation Methods

Specimens with BUB and PED numbers were documented on a Keyence VHX-6000 digital microscope (Keyence, Osaka, Japan). Amber pieces were evened out using a drop of glycerol and a coverslip. Composite imaging (fusing of stacks, merging to panoramas, HDR) was applied with the original built-in software of the microscope.

One specimen (PED 1360) was additionally documented on a Keyence BZ-9000 inverse fluorescence microscope (Keyence, Osaka, Japan). Composite images were assembled with CombineZP (open source) and Adobe Photoshop CS3 (Adobe, San José, CA, USA).

Specimen NIGP209583 was documented on a Zeiss Discovery V16 stereo microscope (Zeiss, Oberkochen, Germany). Helicon Focus 7.0.2 stacking software (Helicon Soft, Kharkiv, Ukraine) was used to combine several images, overcoming limitations in depth of field.

All images were subsequently processed in Adobe Photoshop CS2. This processing included optimisation for colour (histogram), sharpness, and saturation.

### 2.3. Measurements

We measured the relative seta length of the specimens provided here for comparison with extant click beetle larvae. Specimens were measured from literature sources, from images retrieved from the database bugguide.net (accessed on 25 January 2026), and from our own images (full information in Supplementary Table S1), using the measure function in Inkscape (version 1.1; open source) and FIJI (open source). The plots were generated in R [43], using the package ggplot2 (ver. 4.0.0 [44]), and later processed in Adobe Photoshop CS2.

## 3. Results

### 3.1. Morphology of the New Larvae

**Figure 2:**
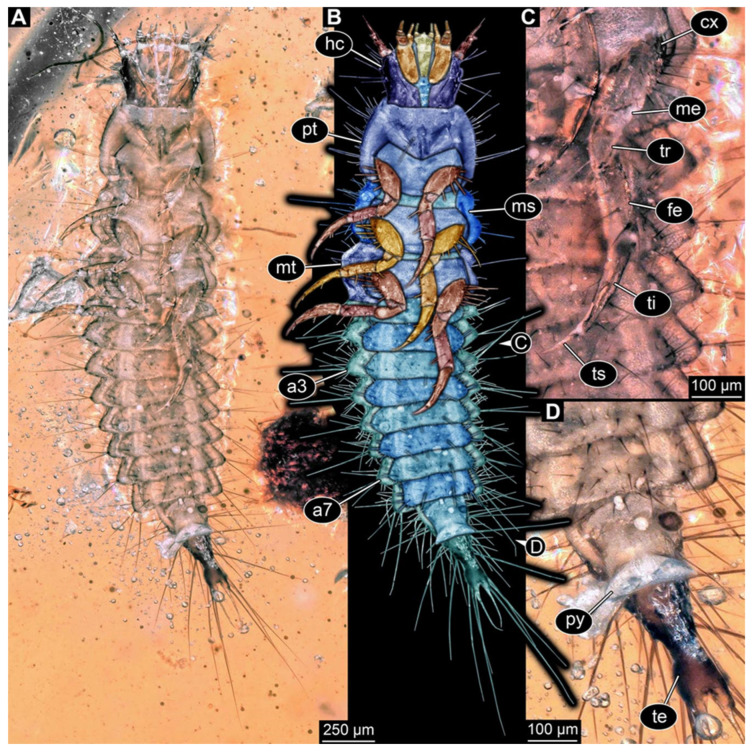
New hairy click beetle larva from Kachin amber, PED 1360, ventral view. (**A**) Overview. (**B**) Colour-marked version of (**A**). (**C**) Close-up on hindleg. (**D**) Close-up on posterior trunk region. Abbreviations: a3–7 = abdomen segments 3–7; cx = coxa; fe = femur; hc = head capsule; me = membrane; ms = mesothorax; mt = metathorax; pt = prothorax; py = pygopod; te = trunk end; ti = tibia; tr = trochanter; ts = tarsungulum.

**Figure 3:**
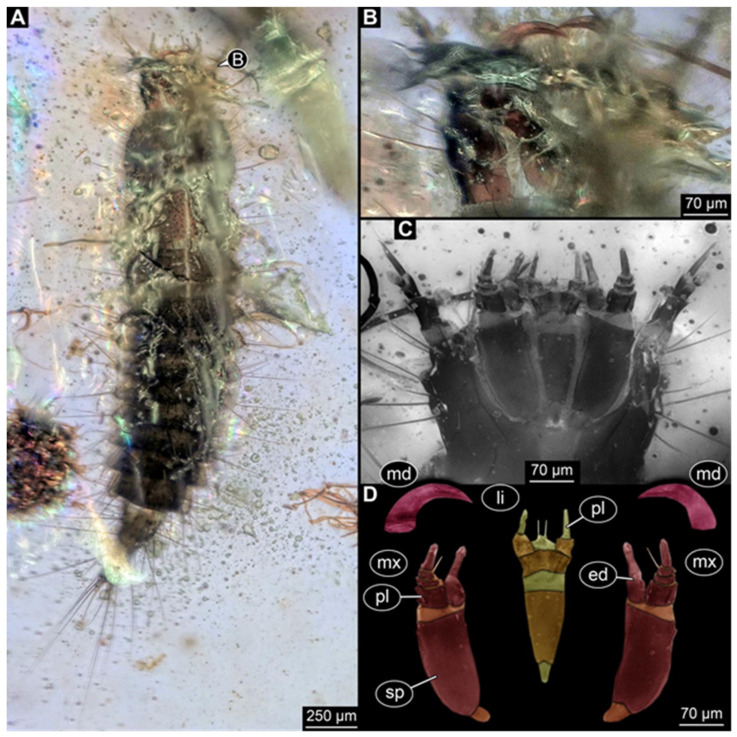
New hairy click beetle larva from Kachin amber, PED 1360, continued. (**A**,**B**) Dorsal view: (**A**) overview; (**B**) close-up on head (note moulting suture). (**C**,**D**) Mouthparts: (**C**) composite-fluorescence micrograph; (**D**) colour-marked mouthparts extracted from (**C**). Abbreviations: ed = endite; li = labium; md = mandible; mx = maxilla; pl = palp; sp = stipes.

**Figure 4:**
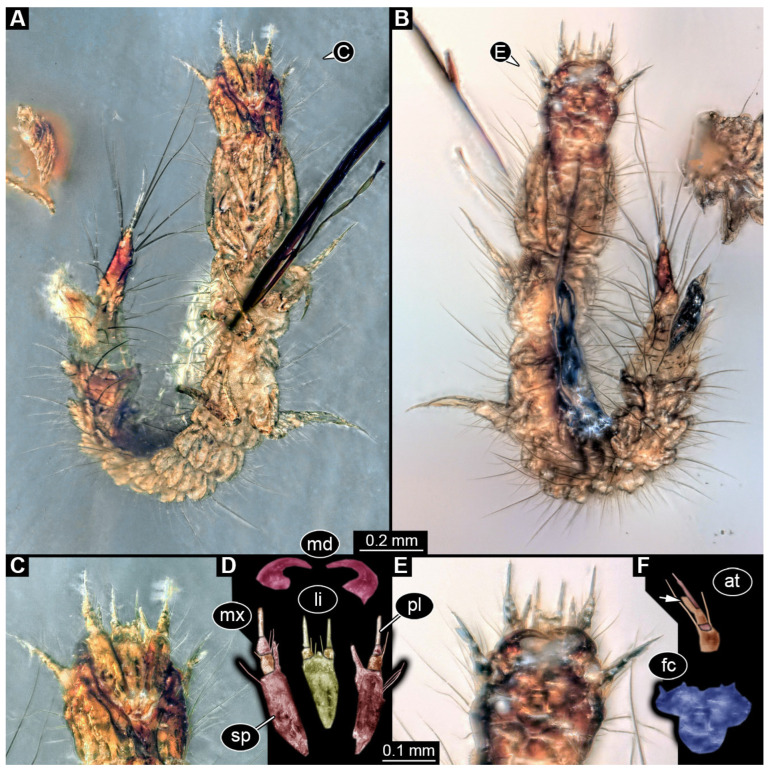
New hairy click beetle larva from Kachin amber, BUB 3071. (**A**,**B**) Overview: (**A**) ventral view; (**B**) dorsal view. (**C**) Head, ventral view. (**D**) Colour-marked mouthparts extracted from (**C**). (**E**) Head, dorsal view. (**F**) Antenna with sensorium (arrow) and fronto-clypeo-labrum (anterior region of head capsule, separated by moulting line) colour-marked, extracted from (**E**). Abbreviations: at = antenna; fc = fronto-clypeo-labrum; li = labium; md = mandible; mx = maxilla; pl = palp; sp = stipes.

**Figure 5:**
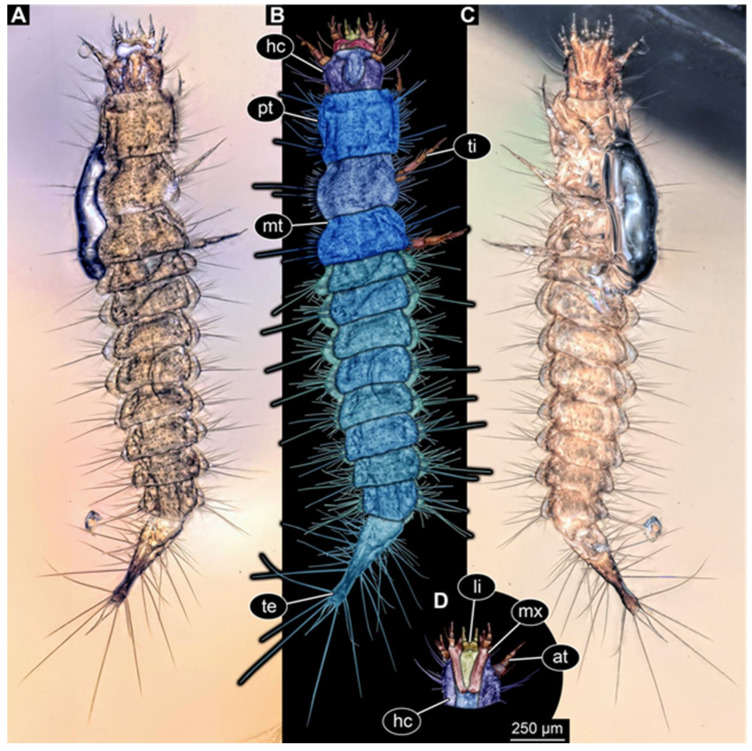
New hairy click beetle larva from Kachin amber, BUB 3087a. (**A**–**C**) Overview: (**A**) dorsal view; (**B**) colour-marked versions of (**A**); (**C**) ventral view. (**D**) Colour-marked head region extracted from (**C**). Abbreviations: at = antenna; hc = head capsule; li = labium; mt = metathorax; mx = maxilla; pt = prothorax; te = trunk end; ti = tibia.

**Figure 6:**
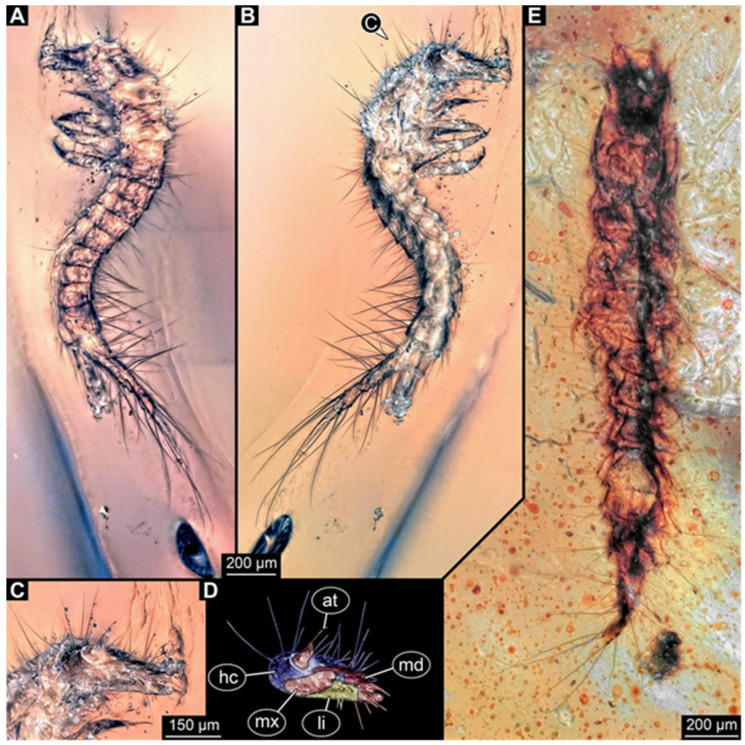
New hairy click beetle larvae from Kachin amber. (**A**–**D**) BUB 3087b. (**A**,**B**) Overview in lateral views: (**A**) left side; (**B**) right side. (**C**) Close-up on head. (**D**) Colour-marked version of (**C**). (**E**) PED 3775, dorsal view. Abbreviations: at = antenna; hc = head capsule; li = labium; md = mandible; mx = maxilla.

**Figure 7:**
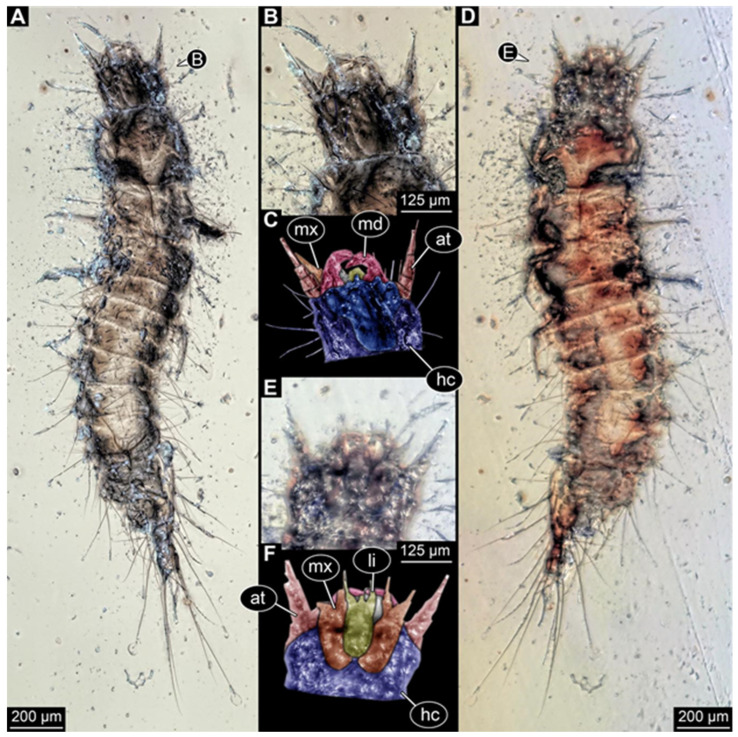
New hairy click beetle larva from Kachin amber, PED 2456. (**A**–**C**) Dorsal view: (**A**) overview; (**B**) close-up on head; (**C**) colour-marked version of (**B**). (**D**–**F**) Ventral view: (**D**) overview; (**E**) close-up on mouthparts; (**F**) colour-marked version of (**E**). Abbreviations: at = antenna; hc = head capsule; li = labium; md = mandible; mx = maxilla.

**Figure 8:**
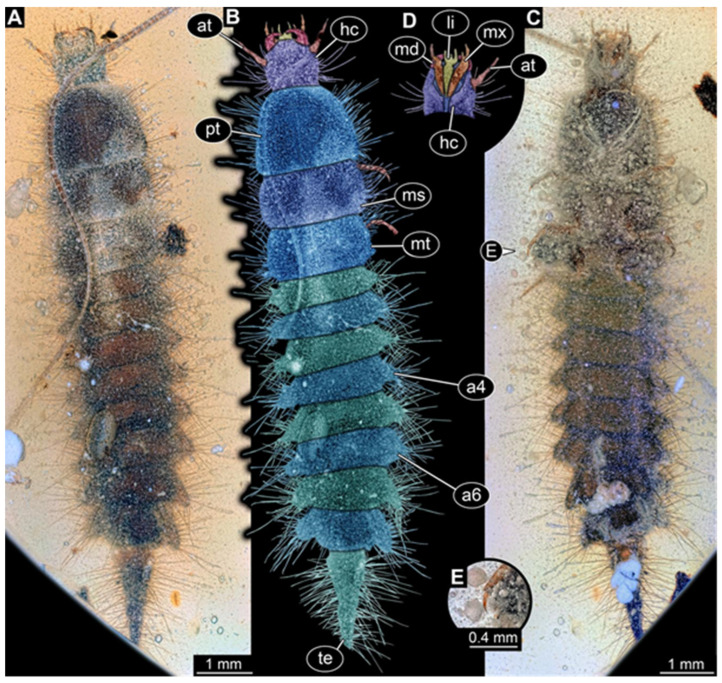
New hairy click beetle larva from Kachin amber, BUB 3692. (**A**,**B**) Dorsal view: (**A**) overview; (**B**) colour-marked version of (**A**). (**C**,**D**) Ventral view: (**C**) overview; (**D**) colour-marked version of head region from (**C**). (**E**) Close-up on tarsungulum. Abbreviations: a4–6 = abdomen segments 4–6; at = antenna; hc = head capsule; li = labium; md = mandible; ms = mesothorax; mt = metathorax; mx = maxilla; pt = prothorax; te = trunk end.

**Figure 9:**
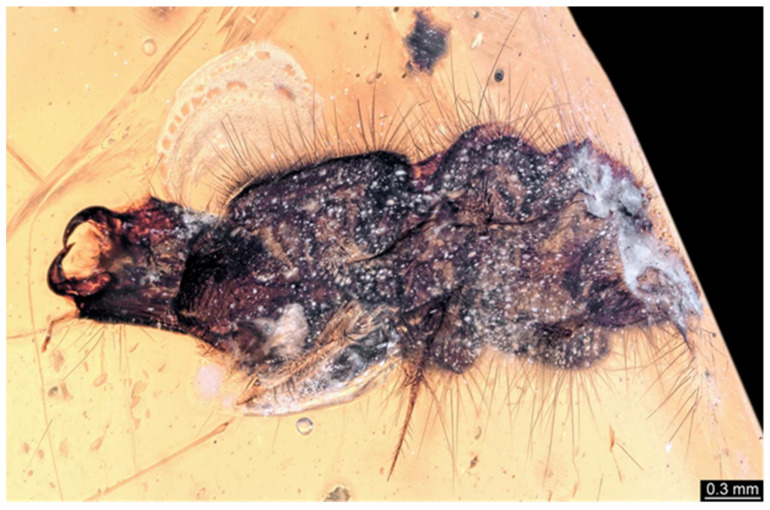
New hairy click beetle larva from Kachin amber, PED 3641, ventral view.

**Figure 10:**
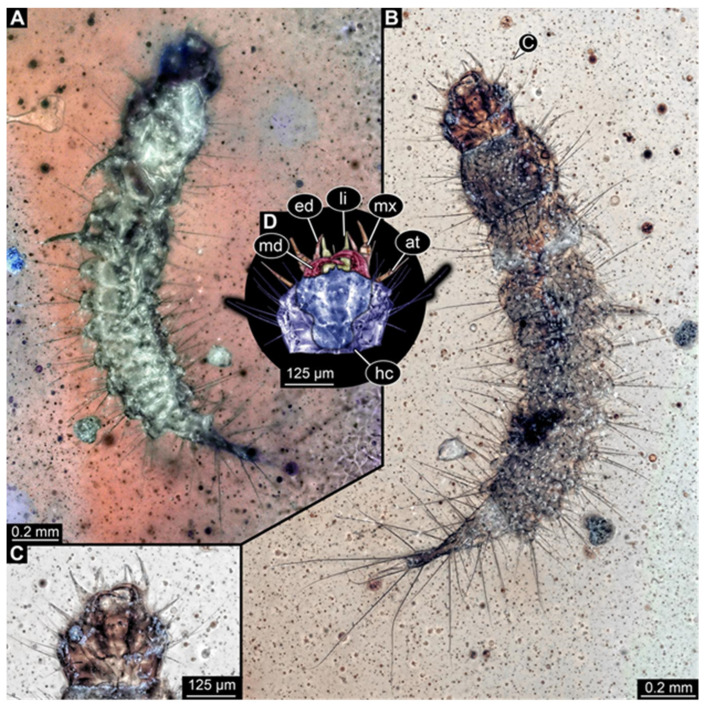
New hairy click beetle larva from Kachin amber, PED 4078. (**A**,**B**) Overview: (**A**) ventral view; (**B**) dorsal view. (**C**) Close-up on head in dorsal view. (**D**) Colour-marked version of (**C**). Abbreviations: at = antenna; ed = endite; hc = head capsule; li = labium; md = mandible; mx = maxilla.

**Figure 11:**
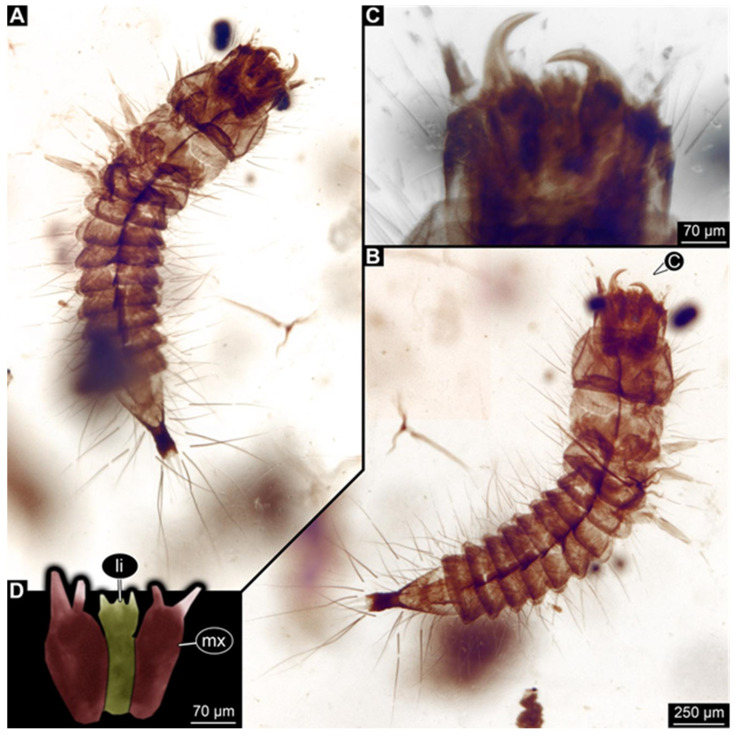
New hairy click beetle larva from Kachin amber, NIGP209583. (**A**) Dorsal view. (**B**–**D**) Ventral view: (**B**) overview; (**C**) close-up on head; (**D**) colour-marked mouthparts extracted from (**C**). Abbreviations: li = labium; mx = maxilla.

**Figure 12:**
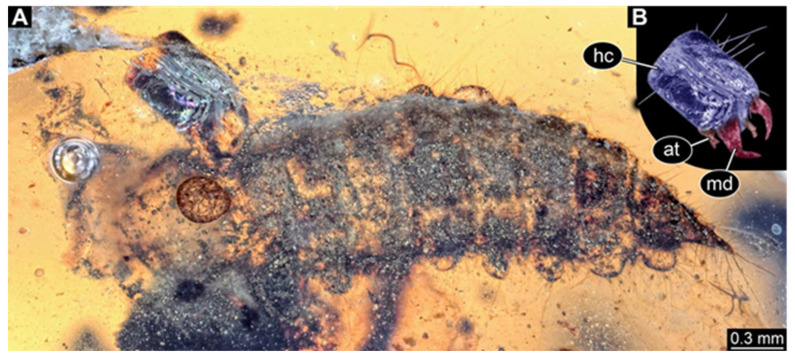
New hairy click beetle larva from Kachin amber, PED 2597: (**A**) overview; (**B**) colour-marked head structures extracted from (**A**). Abbreviations: at = antenna; hc = head capsule; md = mandible.

**Figure 13:**
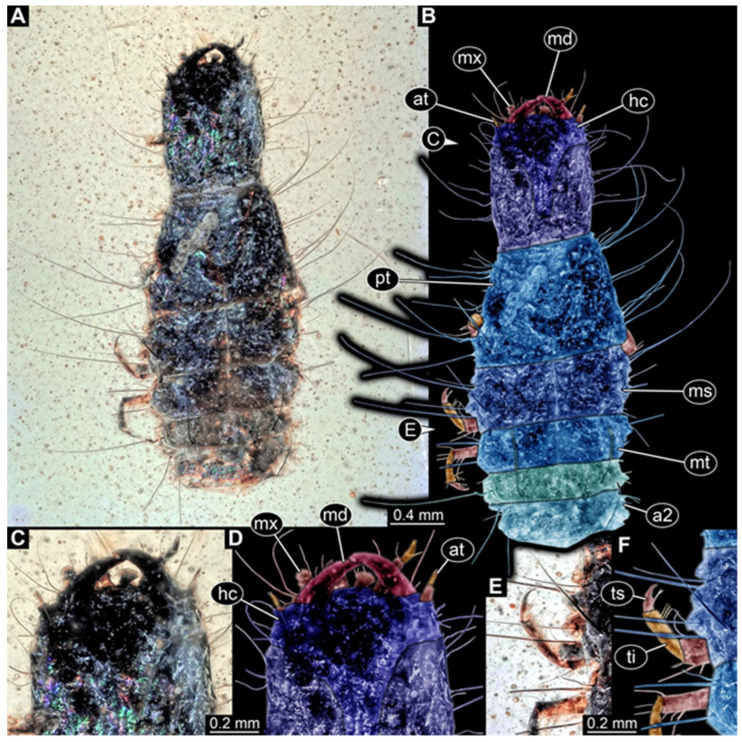
New hairy click beetle larva from Kachin amber, BUB 3707, dorsal view. (**A**) Overview. (**B**) Colour-marked version of (**A**). (**C**) Close-up on anterior head region. (**D**) Colour-marked version of (**C**). (**E**) Close-up on leg. (**F**) Colour-marked version of (**E**). Abbreviations: a2 = abdomen segment 2; at = antenna; hc = head capsule; md = mandible; ms = mesothorax; mt = metathorax; mx = maxilla; pt = prothorax; ti = tibia; ts = tarsungulum.

We found twelve specimens that resemble the larva depicted in Xia et al. [15] (p. 117 bottom left; schematic interpretation in Figure 1). The new specimens have a body with a distinct head with six segments and forward-projecting mouthparts (Figure 2A,B, Figure 3A, Figure 4A,B, Figure 5A–C, Figure 6A–E, Figure 7A,D, Figure 8A–D, Figure 9, Figure 10A–D, Figure 11A–C, Figure 12A,B and Figure 13A,B), an anterior trunk (thorax) with three segments, and a posterior trunk (abdomen) with eight segments and the trunk end (likely conjoined region of evolutionarily original abdomen segments 9–11) (Figure 2A, Figure 3A, Figure 4A,B, Figure 5A–C, Figure 6A,B,E, Figure 7A,D, Figure 8A–C, Figure 10A,B and Figure 11A,B).

The head capsule has a distinct moulting suture (frontal suture), separating the anterior region (fronto-clypeo-labrum) from the posterior one (Figure 3B, Figure 4E,F, Figure 7B,C, Figure 10C,D and Figure 13C,D). The fronto-clypeo-labrum has a distinct backward-oriented, spoon-shaped projection. The ocular segment forms part of the fronto-clypeo-labrum, no prominent stemmata (simple eyes) are apparent, and it is unclear whether this is due to preservation or true absence.

The antennae (appendages of post-ocular segment 1) consist of three elements (antennomeres). The penultimate element has a lateral protrusion (sensorium or sensorial appendix; Figure 3C and Figure 4F). The intercalary segment (post-ocular segment 2) has no externally visible structures.

The mandibles (appendages of post-ocular segment 3) are prominent, simple, and sickle-shaped without apparent teeth (Figure 3B,D, Figure 4D,E and Figure 12B). The maxillae and the labium (appendages of post-ocular segments 4 and 5) together form the maxillo-labial complex (Figure 5D, Figure 7E,F, Figure 8D and Figure 11C,D). The maxillae have an elongated, roughly triangular part (small cardo, prominent stipes), functionally anteriorly bearing a distinct elongate endite (possible galea). Distally, they bear a palp with four elements (Figure 3C,D and Figure 4C,D). The labium is positioned anterior to the cardo of the maxilla. The proximal part has several distinct sclerites: it is elongate and triangular, pointing backwards. Functionally antero-laterally, it has a pair of distinct palps (one on each side), with two elements (Figure 3C,D and Figure 4C,D). Functionally antero-medially, it has a single protrusion (ligula) with a pair of distinct setae (Figure 3C,D).

The three thorax segments each bear a pair of walking legs ventrally (Figure 2C). On thorax segment 1 (prothorax) the legs arise from a distinct set-off posterior region (sclerite?). The segment is longer than the further posterior ones due to the set-off anterior region. Thorax segments 2 and 3 (meso- and metathorax) are similar in structure to the posterior region of the prothorax. Each leg is composed of five units: coxa (basipod), trochanter (endopod element 1), femur (endopod element 2), tibia or tibiotarsus (endopod element 3 or 3 + 4), and tarsungulum or claw (Figure 8E and Figure 13E,F). There is a distinct lateral membraneous area between coxa and trochanter (Figure 2C).

Eight abdomen segments bear distinct tergites (Figure 3A). Ventrally, each of these abdomen segments bears a distinct sclerite. The pleural membrane of each segment protrudes laterally. The abdomen segments become consecutively narrower towards the posterior end.

The trunk end is triangular, tapering posteriorly; the very posterior tip is widening again, forming a slightly forked end (Figure 2D). Ventrally on the trunk end, the anal region forms a distinct pygopod (Figure 2D and Figure 6A,B).

The entire body bears prominent setae. Especially long setae arise from the drawn-out regions of the pleural membranes and the forked tip of the trunk end.

### 3.2. Differences

**Figure 14:**
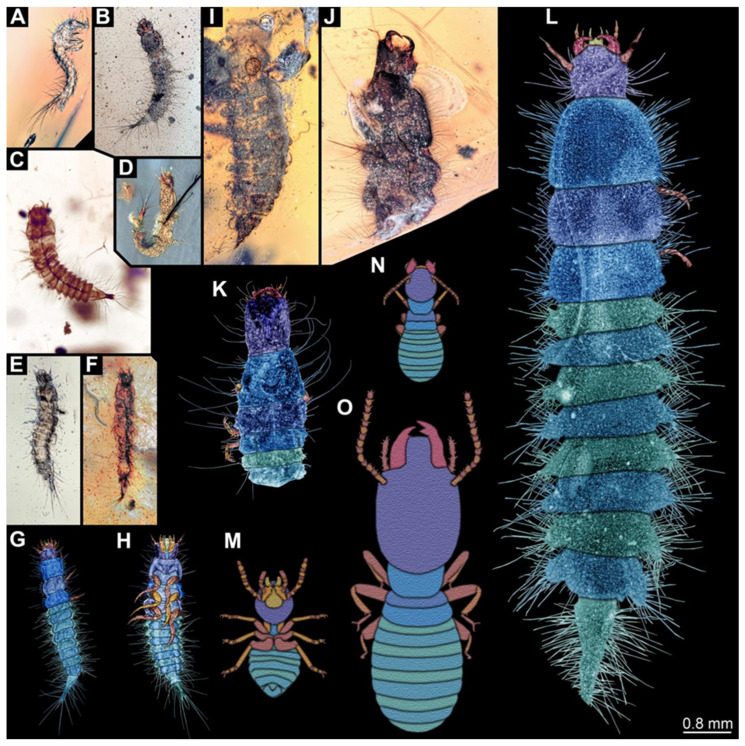
Size comparison of the hairy click beetle larvae and termites from Kachin amber. (**A**–**L**) New larvae, all to the same scale: (**A**) BUB 3087b; (**B**) PED 4078; (**C**) NIGP209583; (**D**) BUB 3071; (**E**) PED 2456; (**F**) PED 3775; (**G**) BUB 3087a; (**H**) PED 1360; (**I**) PED 2597; (**J**) PED 3641; (**K**) BUB 3707; (**L**) BUB 3692. (**M**–**O**) Termites, simplified from [45].

Most specimens strongly resemble each other, but a major factor in which they differ is their body size (Figure 14A–L). However, some specimens also differ in certain other characteristics, while sharing the overall morphology. In specimen BUB 3707 (Figure 13), the head capsule is more elongate than in most other specimens. A similar morphology occurs in PED 2597 (Figure 12). In the latter specimen, the trunk also appears stouter, especially the trunk end. In specimen BUB 3707, this region is not preserved.

### 3.3. Seta Length

**Figure 15:**
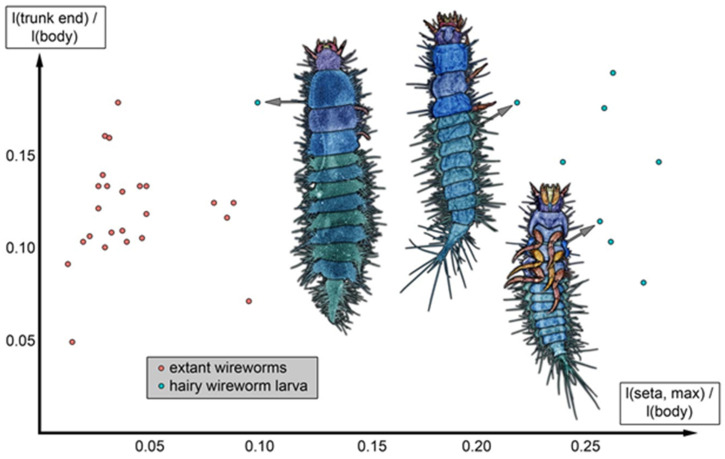
Scatterplot of relative length of trunk end vs. relative maximum seta length (both calculated vs. overall body length). Note that in the new fossil larval type (hairy click beetle larva), the setae are quite long; the setae are relatively shortest in the largest specimen.

Plotting the relative length of the trunk end (divided by body length) versus the relative length of the longest seta (also divided by body length) reveals that the fossils have rather long setae compared to many extant click beetle larvae (Figure 15).

## 4. Discussion

### 4.1. Identity of the Specimens: Click Beetle Larvae

All specimens resemble each other to a high degree and also resemble the specimen reported by Xia et al. [15] (p. 117 bottom left; figure 1). The overall habitus of the specimens clearly identifies them as holometabolan larvae. The campodeiform appearance and the arrangement of the mouthparts identify these animals as beetle larvae.

The details of the mouthparts allow us to reach a further conclusion. The maxillae reach slightly behind the labium, hence forming a functional maxillo-labial complex [46]. The exact arrangement is very characteristic of larvae of Elateridae. The strongly triangular labium is well known, for example, in larvae of Agrypninae. Another characteristic of Elateridae is the vase-shaped (lyriform) moulting suture of the head capsule [5], well observable in some of the fossils. Further characteristics compatible with the larvae of Elateridae are a sensorial process on the penultimate element of the antenna, as well as the anal membrane being developed as a pygopod. It is therefore highly likely that these fossil larvae are click beetle larvae. As most of the specimens strongly resemble each other, it seems likely that they represent a single species or several closely related species with a common larval morphotype.

Specimen PED 2597 differs from the other specimens in the relative length of the trunk end. It also appears bulkier overall, the setae are less prominent, and the head is slightly more elongate. However, the specimen is not well preserved; the head is even detached. It is unclear if it was strongly mangled or if it represents an exuvia. The latter interpretation could explain how the head became detached. The differences to most of the other specimens may be related to the worse overall preservation of specimen PED 2597.

Specimen BUB 3707 differs slightly more from the other specimens. The head appears more elongate than in the other specimens (in this aspect resembling PED 2597). While it has very long setae, comparable to the other specimens, there seem to be fewer such setae present (difference also to PED 2597). Further posterior structures are not preserved and cannot be compared. Still, the similarities appear sufficient to us to discuss all specimens together.

### 4.2. Possible Relationships Within Elateridae

The new type of larva presents some peculiarities unexpected for a click beetle larva. In what follows, we will discuss how far such morphological features are in accordance with the larval type being interpreted as a click beetle and further explore the possible relationships of the new larval type within Elateridae. We will discuss the most intriguing morphological aspects in detail. However, the phylogenetic relationships and taxonomic interpretations of major ingroups of Elateridae have changed quite drastically over the years [47,48,49], particularly in the last decade (e.g., Ref. [50] vs. Refs. [51,52,53,54,55]), rendering character reconstructions challenging. There is also a significant lack of knowledge about the larvae for several ingroups of Elateridae [56,57,58], making it possible that any larval morphology supposedly specific to an ingroup is, in fact, more common.

#### 4.2.1. Body Shape

The “typical” wireworm is cylindrical to sub-cylindrical (Ref. [5] p. 411; Ref. [59] figures 1–3, p. 291), as in many representatives of Elaterinae (Ref. [60] figures 6 and 7, p. 14; Ref. [61] figure 34.452a, p. 418). However, the new type of larva has a rather flattened body. Such a morphology has also been reported for different modern-day click beetle larvae, such as representatives of Lissominae (*Austrelater*: Ref. [62] p. 1352) or Agrypninae (Hemirhipini: Ref. [63] p. 704; Ref. [64] p. 94; Pyrophorini: Ref. [65] figures 6–8, p. 29).

#### 4.2.2. Pleural Membrane

**Figure 16:**
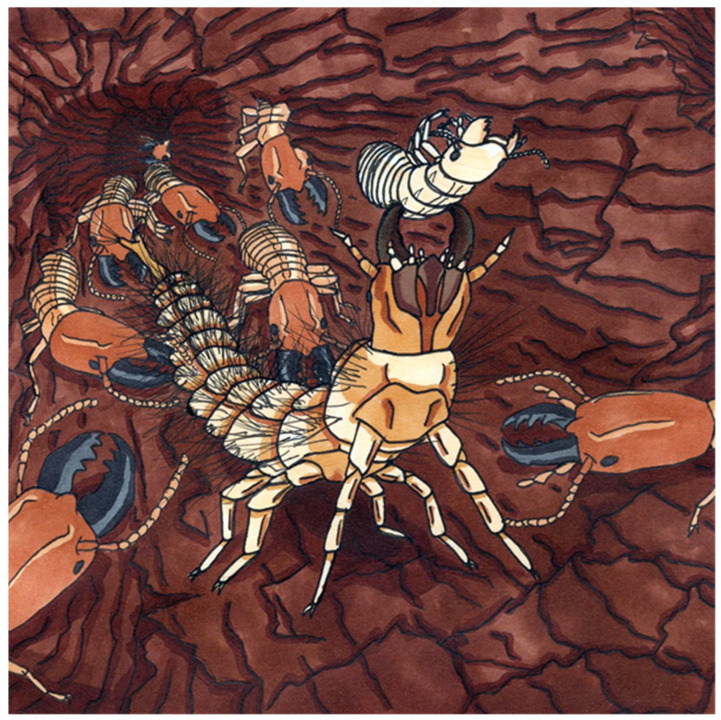
Palaeoartistic interpretation of the new hairy click beetle larva preying in a termite nest, prepared by one of the authors (GTH).

Especially in the cylindrical forms, but also in the more flattened ones, extant larvae present only the tergites in dorsal view; no pleural membrane is visible. This is the case for most ingroups, for example, in the larvae of Lissominae (*Austrelater*: Ref. [62] figure 29, p. 1362), Elaterinae (*Ischiodontus*: Ref. [60] figures 6 and 7, p. 14), Agrypninae (Pyrophorini: Ref. [65] figures 6–8, p. 29) or Dendrometrinae (*Ctenicera*: Ref. [66] figures 10 and 16, pp. 72, 73; *Athous*: Ref. [67] pl. V, figure 4a).

In the fossils, the pleural membrane is very apparent and even appears to be bulging. Also, this condition can be found in certain extant larvae (although not as strongly expressed), more precisely within Agrypninae, for example, in Hemirhipini ([63] p. 705, figure 1; Ref. [64] figure 30, p. 103) or Platycrepidiini ([68] figure 4, p. 322).

#### 4.2.3. Trunk End

The common type of trunk end in the larvae of Elateridae is a short and bifid or forked one. This type is, for example, found in the extant larvae of Lissominae (*Austrelater*: Ref. [62] figure 29, p. 1362), Agrypninae (diverse ingroups: Ref. [63] p. 705, figure 1; Ref. [64] figure 30, p. 103; Ref. [65] figures 6–8, p. 29; Ref. [68] figure 4, p. 322; Ref. [69] figure 1, p. 349; Ref. [70] figure 8 left, p. 7; Ref. [71] figure 1A, p. 637; Ref. [72] figure 2, p. 2; Ref. [73] figure 1A, p. 303; Ref. [74] figure 2, p. 1056; Ref. [75] figure 2D, p. 35) or Dendrometrinae (*Ctenicera*: Ref. [66] figure 13, p. 72; *Athous*: Ref. [67] pl. V, figure 4b). In few groups, the trunk end is more elongated, as seen in the fossils, e.g., in Elaterinae, but the trunk end is then either more rounded (Physorhinini: Ref. [59] figures 1–3, p. 291) or triangular but without a bifid tip, as is present in the fossils (*Ischiodontus*: Ref. [60] figures 6 and 7, p. 14; *Athous*: Ref. [67] pl. V, figure 3a,c). The highest similarity to the new fossils concerning the trunk end appears to occur in certain larvae of Omalisinae ([76] figure 9 colour, pl. 5). However, these larvae differ significantly from the new fossils in their elongated mouthparts ([76] figures 3–8, colour pl. 4).

#### 4.2.4. Long Setae

In most larvae of Elateridae, there are only few very short setae arising from the body, unlike in the fossils. This is the case, for example, in Lissominae (*Austrelater*: Ref. [62] figure 29, p. 1362), Elaterinae (Ref. [59] figures 1–3, p. 291; Ref. [60] figures 6 and 7, p. 14), or Agrypninae (various ingroups: Ref. [63] p. 705, figure 1; Ref. [64] figure 30, p. 103; Ref. [65] figures 6–8, p. 29; Ref. [68] figures 4 and 12, pp. 322, 324; Ref. [69] figure 1, p. 349; Ref. [71] figure 1A, p. 637; Ref. [72] figure 2, p. 2; Ref. [73] figure 1A, p. 303). In particular, many larvae of Drilini (ingroup of Agrypninae) are very hairy (e.g., Ref. [77] figure 4, p. 168; Ref. [78] figure 7, p. 9). However, the body shape of these larvae differs significantly from the larvae described here. It therefore seems unlikely that the new larvae are representatives of Drilini. In some species of Tetralobinae, the sister group of Agrypninae [53], the larvae have long and numerous setae (Ref. [70] figure 8 left, p. 7). Yet, in other respects, these larvae do not resemble the new fossils. Interestingly, the recently reported larvae of Elateridae from Myanmar amber, identified as possible representatives of Pityobiinae, also had relatively long setae in comparison to their modern counterparts, although not as long as in the larvae reported here [3,40]. Due to this similarity, the new larvae may also be specialised representatives of Pityobiinae, which share several similarities with the larvae of Agrypninae and Dendrometrinae (see also [40]).

Overall, the fossils have certain characteristics known from different ingroups of Elateridae, but no modern larvae show the combination seen in the fossils. In any interpretation, this character distribution points to cases of convergent evolution. For example, long setae, a slightly flattened body, and bulging pleural membrane are all characteristics compatible with a position in Agrypninae (taking a simplified view, because within Agrypninae, these characteristics are also scattered). However, in this case, it has to be assumed that the rather elongate trunk end, with its triangular shape, evolved convergently to that in Elaterinae and also in Omalisinae. Convergent evolution does not seem to be unusual within Elateroidea (see discussion in [27]). The rapid diversification of lineages should lead to rather similar-appearing animals, as they would have been facing similar selective pressures. Therefore, convergence should not be a surprising phenomenon in species-rich lineages (see also [3,40]).

Despite these uncertainties, the available characteristics support an interpretation of the new larvae being representatives of Elateridae. This represents only the fourth type of click beetle larvae from Kachin amber (first: Ref. [38]; second: Refs. [3,40]; third: Ref. [39]). There have, to date, only been a few adult click beetles described from Kachin amber, which are representatives of Agrypninae, Dendrometrinae, Elaterinae, or Pityobiinae, or could not be identified further than to Elateridae [79,80]. The newly reported larvae may represent immatures of some of these species (especially Agrypninae or Pityobiinae, as discussed above), yet this cannot be further substantiated without syninclusions of pupae and the preceding and subsequent ontogenetic stages.

### 4.3. Lifestyle of the New Fossil Larval Type

As the new larval type does not immediately resemble any of the modern click beetle larvae, an interpretation of its lifestyle is more difficult than for cases in which we have directly matching modern-day counterparts. The mouthpart shape, especially the sickle-shaped mandibles, argues for a predatory lifestyle; mandibles of click beetle larvae feeding on plants are quite differently shaped (e.g., Ref. [81] figure 4, p. 133; Ref. [82] figure 3b, p. 5). The strongly worm-shaped extant larvae are highly flexible and can, therefore, enter confined spaces, for example, when hunting wood-boring larvae of other beetles. Such a lifestyle also explains the rather short setae in most of the extant larvae. However, the larvae with long setae can also live in confined spaces [70], for example, in termite nests [83,84,85,86]. That they lived in termite nests is a possible interpretation of the new fossil larvae, with these nests being associated with wood, which makes preservation in amber more probable. In this respect, the setae may be of further interest. Predators of eusocial insects face severe dangers as, unlike when attacking a solitary individual, numerous individuals will react to an attack. Termites are indeed known to be found in Kachin amber [87,88], and they already have well differentiated soldier morphs in the Kachin amber forest [45].

Setae can have different functions, but they are well known to provide a defensive function, for example, for caterpillars [89] or some beetle larvae [90]. There are also predatory caterpillars, and some even attack nests of eusocial aphids. For these, it has been demonstrated that the setae fend off the soldiers. A simple size comparison of the new larvae with known termite soldiers reveals that the setae of the larvae would indeed represent a barrier, making it more difficult for the soldiers to reach the larvae (Figure 14). However, this possible interaction remains speculative (Figure 16), as none of the fossils were preserved together with a termite. Still, this case further indicated that long setae may be advantageous for a predatory larva.

### 4.4. Diversity of Beetle Larvae in Kachin Amber

As of now, beetle larvae in Kachin amber are still under-represented in comparison to other holometabolan larvae, e.g., of lacewings [3]. The new morphotype adds to this diversity. It is, furthermore, one of the few cases in which the fossil larvae might outperform most of their extant close relatives. In the Cretaceous fauna, several other neuropteriformian larvae with extreme morphologies were present, which are unparalleled in the modern fauna, indicating a loss in diversity [91]. In beetle larvae, it seems that the modern fauna is as diverse or even more diverse than it was in the Cretaceous [28,39]. Only a few finds indicate that we might have lost some beetle larval types after the Cretaceous (e.g., Ref. [39]). The unusual character combination of the here-reported larvae makes them a candidate for such a case. Interestingly, in another beetle lineage, namely, Dermestidae, the length of the setae also provided similar signal of a possible loss [90].

## Data Availability

All data from this study are available in this paper and associated papers.

## Supplementary Materials

The following supporting information can be downloaded at https://www.mdpi.com/article/10.3390/insects17030271/s1: Supplementary Table S1: Information on the measured specimens and ratios of the measurements (maximal seta length/body length; trunk end length/body length). Figure 2, Figure 3, Figure 4, Figure 5, Figure 6, Figure 7, Figure 8, Figure 9, Figure 10, Figure 11, Figure 12 and Figure 13 in high resolution are available at https://doi.org/10.5281/zenodo.18250375 (accessed on 22 January 2026).
